# Experimental Investigation of Bacterial Inactivation of Beef Using Indirect Cold Plasma in Cold Chain and at Room Temperature

**DOI:** 10.3390/foods13172846

**Published:** 2024-09-07

**Authors:** Peiru Li, Hainan Zhang, Changqing Tian, Huiming Zou

**Affiliations:** 1 Technical Institute of Physics and Chemistry, Chinese Academy of Sciences, Beijing 100190, China; lipeiru19@mails.ucas.edu.cn (P.L.); chqtian@mail.ipc.ac.cn (C.T.); zouhuiming@mail.ipc.ac.cn (H.Z.); 2 University of Chinese Academy of Sciences, Beijing 100049, China; 3 Key Laboratory of Cryogenic Science and Technology, Beijing 100190, China; 4 School of Energy and Environmental Engineering, University of Science and Technology Beijing, Beijing 100083, China

**Keywords:** cold plasma, cold chain, beef, bacterial inactivation

## Abstract

Pathogen contamination is a severe problem in maintaining food safety in the cold chain. Cold plasma (CP) is a novel non-thermal disinfection method that can be applied for the bacterial inactivation of food in appropriate contexts. Currently, research on CP used on food at cold chain temperatures is rare. This work investigated the bacterial inactivation effect of CP on beef at typical cold storage temperatures of 4 and −18 °C and room temperature (25 °C). The reactive species in CP were indirectly tested by evaluating O_3_, NO_3_^−^ and NO_2_^−^ in cold plasma-activated water (PAW), which indicated the highest concentrations of reactive species in CP at 25 °C and the lowest at −18 °C. The bactericidal efficacy of CP treatment against beef inoculated with *Escherichia coli* at −18 °C, 4 °C, and 25 °C was 30.5%, 60.1%, and 59.5%, respectively. The 4 °C environment was the most appropriate treatment for CP against beef, with the highest bactericidal efficacy and a minor influence on beef quality. The indirect CP treatment had no significant effect on the texture, color, pH, or cooking loss of beef at −18 °C. CP shows significant potential for the efficient decontamination of food at cold chain temperatures.

## 1. Introduction

Challenges related to potential pathogenic contamination exist throughout processing, distribution, retail, and preparation in the meat industry. The contamination of meat could lead to foodborne disease outbreaks [1,2]. In previous stages under typical refrigerated (4 °C) and frozen storage (−18 °C), pathogens such as *Listeria monocytogenes*, *Aeromonas hydrophila,* and *Staphylococcus aureus* have been shown to survive on food and its packaging [3,4]. The pathogen contamination of frozen meat products is frequently reported [5,6], posing a risk for public health. In addition, the ice and low temperatures in the cold chain environment cause problems for current disinfection methods, such as the reduced chemical efficacy of disinfects, physical barriers due to ice, the malfunction of equipment at low temperatures, and increased microbial resistance [7]. Therefore, the typical cold chain environment requires a safe and effective bacterial inactivation method.

In the past, many bacterial inactivation methods have been proposed to reduce pathogen contamination in food. These methods include ultraviolet [8], irradiation [9], chemical disinfectants [10], and cold plasma (CP) [11]. Among these, CP treatment is an innovative method showing promising effects in bacterial inactivation [12]. Plasma is a partially ionized gas containing free radicals, ions, electrons, excited and ground-state molecules, and ultraviolet radiation, categorized into thermal plasma and non-thermal plasma, i.e., CP. CP is characterized by a high temperature of light electrons, while other species, such as molecules and heavy atoms, remain close to room temperature. It is in a thermodynamic non-equilibrium; thus, its temperature is lower than that of thermal plasma [13,14,15,16,17]. Although the power source of CP often requires a high voltage, the energy consumption is lower compared to thermal processing due to the rapid breakdown of the dielectric medium and the low current requirements [18,19].

CP has the advantages of a low operating temperature, minimal energy consumption, effective bacterial inactivation, and no residues [15,16]. Therefore, it is favorable for use in food processing in low-temperature environments. CP is generated with air or inert gas under atmospheric or lower pressure. The methods of generating CP include dielectric barrier discharge (DBD), plasma jet corona discharge, radiofrequency plasma generation, and microwave plasma generation [16]. The high efficiency and low maintenance costs of DBD, together with easy access to air, make air-based DBD CP appealing for industrial use.

Several studies have investigated the inactivation effect of CP against various microorganisms and pathogens on food materials [11,20,21,22,23,24,25,26]. Several explanations for the bacterial inactivation effect of CP have been proposed, although it remains poorly understood. First, reactive oxygen species (ROS), reactive nitrogen species (RNS), and energized ions in the CP inactivate the pathogenic microorganism by oxidating the DNA, proteins, and lipids of cells and attack the intracellular components. Then, the charged particles in CP inactivate the bacteria by electroporation [24,27].

Although the average temperature of CP is around room temperature, the direct treatment of air plasma might create hot spots on the sample under circumstances such as a high power density, the uneven distribution of CP, and rough sample surfaces, resulting in the degradation of the color and appearance of the food [28]. In this context, the indirect treatment of food with CP, in which the samples are placed outside the discharging area and the reactive species in CP are air-pumped toward the samples, has better application potential [28]. However, the indirect mode of CP treatment is relatively less investigated. Furthermore, the bacterial inactivation effect and physical parameters of CP under a cold chain environment requires comprehensive investigation prior to its application in this field.

Regarding the application of CP in the precooling of vegetables and fruits, our team has previously researched the effect of combining precooling and cold plasma-activated water (PAW) on the bacterial inactivation of snow peas [29]. To apply CP during meat storage, this research investigates the bacterial inactivation of CP in cold storage environments. In addition, the device for the generation of CP without water is simpler than that used for PAW; thus, it has wider application potential encompassing various food types. Furthermore, the previous literature has not reported the bacterial inactivation effect of indirect CP on food at typical cold chain temperatures (4 and −18 °C).

The aim of this study was to investigate the bacterial inactivation effect of air-based DBD CP on beef at typical cold storage temperatures of 4 and −18 °C and compare it with that at room temperature, i.e., 25 °C. The CP was pumped towards beef in an indirect manner. The reactive species in the CP at different temperatures were indirectly examined by PAW. The influence of indirect CP treatment on the beef property indexes of color, pH, cooking loss, and shear force was evaluated. The influence of indirect cold treatment on the color of the beef was compared to that of direct treatment. This work offers a bacterial inactivation method that maintains the safety and quality of food under a cold chain environment, laying the foundation for the application of indirect CP treatment in cold chain disinfection.

## 2. Materials and Methods

### 2.1. Beef Preparation

Beef tenderloin from a cow slaughtered on the previous day was purchased from the local market in Langfang, Hebei, China. The beef was cut into 2 × 2 × 2 cm pieces with an average weight of 12.9 g. The beef was stored at −18 °C for 24 h to be frozen before CP treatment under a −18 °C environment or a 4 °C environment for 2 h before exposure to CP at 4 °C or 25 °C. In the current study, three samples were used for the bactericidal efficacy test and six samples were used for the quality index test for each treatment to ensure the reproducibility of the results.

### 2.2. Experimental Apparatus

**Figure 1:**
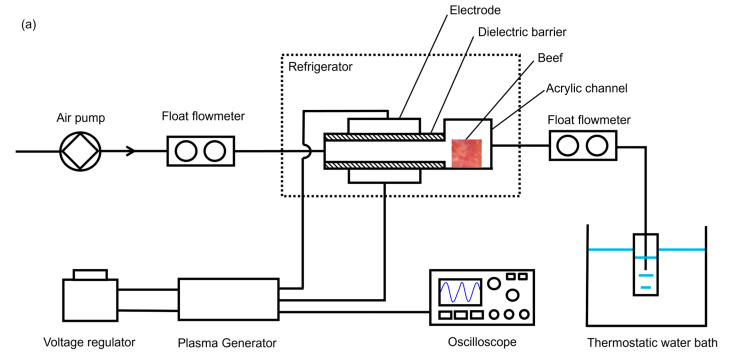

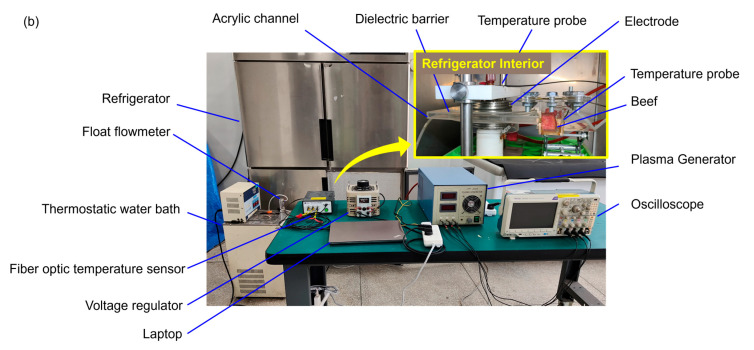
Experimental apparatus for CP bacterial inactivation. (**a**) Schematic diagram; (**b**) experimental devices.

The experimental apparatus used to investigate the bacterial inactivation effect of CP is shown in Figure 1. The apparatus included an air DBD reactor (Nanjing Suman Plasma Technology Co., Ltd., Nanjing, China), a plasma generator (CTP-2000S, Nanjing Suman Plasma Technology Co., Ltd., Nanjing, China), a voltage regulator (Nanjing Suman Plasma Technology Co., Ltd., Nanjing, China), an oscilloscope (MDO3024, Tektronix, Inc., Beaverton, OR, USA), an air pump (ACO-001A, Raoping Xingcheng Aquarium Products Co., Ltd., Raoping, China), a float flowmeter (LZM-4T, Yuyao Jintai Meter Co., Ltd., Yuyao, China), and a thermostatic water bath (DC-0510, Ningbo Scientz Biotechnology Co., Ltd., Ningbo, China).

The DBD reactor consisted of two circular electrodes with a diameter of 98 mm; two 3-mm-thick pieces of circular quartz glass with a diameter of 185 mm, used as dielectric barriers; and an acrylic channel with a thickness of 3 mm. The distance between the two DBD high-voltage electrodes ranged from 0 to 20 mm. The gap between the two electrodes was set to 20 mm for the direct and indirect treatments. The voltage and current of the CP were measured using the oscilloscope with an acquisition frequency of 250 MHz through the voltage and current port of the plasma generator. The voltage port was connected to a capacitor, scaling down the output voltage at the ratio of 1000:1. The current port was connected to a 50 Ω resistor, which produced the signal in voltage form. The measured voltage and current exerted on the electrode are shown in Figure S1 in the Supplementary Materials. The voltage had a differential pattern with a peak of 15 kV. According to Figure S1, the power needed to generate the CP was 1.68 ± 0.09 W, calculated using Equation (1). In Equation (1), *P* is the power, *V* is the voltage, *I* is the current, *t* is the time, and *T* is the period.

(1) P=∫VIdt/T

To evaluate the bacterial inactivation effect of CP at 4 °C and −18 °C, the DBD reactor was placed in a refrigerator (MR-BCD-CFG4MBXG, Qingdao Meilai Intelligent Technology Co., Ltd., Qingdao, China) with a temperature deviation of ±1 °C. To investigate the inactivation effect of CP at room temperature, the reactor was placed in the laboratory at 25 °C ± 1 °C, controlled by an air conditioner.

**Figure 2:**
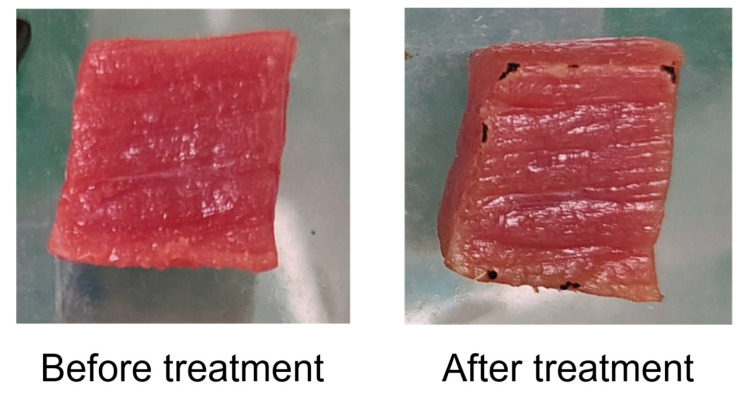
Appearance of beef before and after direct treatment with CP.

The direct treatment with CP was tested first for comparison purposes. Direct treatment with CP was applied to the beef. Specifically, the beef was placed between the electrodes at 17.5 ± 0.5 °C. The color index and appearance of the beef are shown in Table 1 and Figure 2. The results showed that direct treatment with CP significantly decreased the a* and burned the edges of the beef. Therefore, indirect treatment with CP was applied in the current research to investigate the bactericidal effect of indirect CP. The CP generated between the electrodes was pumped through an acrylic channel to the beef samples. The airflow rate was 4 L/min. The treatment time for beef was 4 min.

### 2.3. Detection of Reactive Species in CP

The ROS and RNS in CP can be either directly measured by a Fourier transform infrared spectrometer (FTIR) or indirectly measured by ions in PAW. FTIR can detect transient reactive species in CP. The indirect method can measure the long-lasting reactive species in CP with a simple operation.

In this study, the reactive species in CP were indirectly measured from the O_3_, NO_3_^−^, and NO_2_^−^ in PAW. Exhaust gas was directed into a tube containing 20 mL of distilled water to form PAW, which was immersed in a thermostatic water bath. The concentrations of O_3_, NO_3_^−^, and NO_2_^−^ in the PAW were measured using a UV–Vis spectrophotometer (UV-2355, UNICO (Shanghai) Instrument Co., Ltd., Shanghai, China) according to the Beer–Lambert law [30]. The previously established correlation between the active substances and concentrations at characteristic wavelengths is shown in Table 2. The inlet air temperature was maintained at 25, 4, and −18 °C. The air was pumped through the reactor, reacted between the electrodes, and directed to the water bath. When the inlet air temperature was 25 °C, the water bath temperature was 25 °C. When the inlet air temperature was 4 or −18 °C, the temperature of water bath was adjusted to 4 °C. The temperature of the environment was measured by a fiber optic temperature sensor (Indigo Precision Co., Ltd., Suzhou, China). The probes were placed near the electrode and outer surface of the acrylic channel, as shown in Figure 1b.

### 2.4. Bacteria Preparation

*Escherichia coli* (*E. coli*, ATCC25922), preserved in the laboratory, was chosen as the test strain to evaluate the bacterial inactivation effect of CP. The beef samples were divided into four groups. Samples in group i were inoculated with *E. coli* and were not treated with CP; samples in group ii were inoculated with *E. coli* and were treated with CP; samples in group iii were not inoculated with *E. coli* and were not treated with CP; samples in group iv were not inoculated with *E. coli* and were treated with CP.

For groups i and ii, first, the *E. coli* strain was inoculated from a nutrient agar medium plate into 20 mL trypticase soy broth (Qingdao Hope Bio-Technology Co., Ltd., Qingdao, China). The liquid suspension, sealed with a vented sealed film, was vibrated in a water bath (SHA-B, Changzhou Jintan Liangyou Instrument Co., Ltd., Changzhou, China) at 37 °C and 200 rpm for 24 h. Then, 1 mL of the prepared *E. coli* suspension was serially diluted 10-fold using 9 mL of sterile physiological saline to reach a concentration with a magnitude of 10^8^ CFU/mL. Then, 0.15 mL of the diluted suspension was inoculated onto the samples, either frozen or not. For groups i to iv, after treatment with CP for 240 s or after the same period of time for groups not treated with CP, the samples were placed in a sterile homogeneous bag containing 100 mL of sterile physiological saline. The samples were homogenized in the bag using a homogenizer (HN-08, Shanghai Hannuo Instruments Co. Ltd., Shanghai, China). Then, the pour plate method was applied to determine the CFU in the samples [32]. The suspension was serially diluted using sterile physiological saline. For each dilution, 1 mL of suspension was mixed with 9 mL of sterile physiological saline. From the final three serial dilutions, 1 mL of each diluted suspension was transferred into a separate Petri dish using a pipette. Finally, approximately 15 mL of molten nutrient agar medium (Qingdao Hope Bio-Technology Co., Ltd., Qingdao, China) was poured onto each Petri dish. For groups iii and iv, molten plate count agar (Beijing Land Bridge Co., Ltd., Beijing, China) was used instead. Afterwards, the solidified agar medium was incubated at 37 °C for 24 h. Finally, the viable microbial colonies were counted to calculate the CFU in the beef samples according to Equation (2). The bactericidal efficacy is defined as in Equation (3).

(2) CFU=Total colony number×dilution factor

(3) $$\mathrm{Bactericidal}\;\mathrm{efficacy}=\left(\mathrm{CFU}\;\mathrm{without}\;\mathrm{CP}-\mathrm{CFU}\;\mathrm{with}\;\mathrm{CP}\right)/\mathrm{CFU}\;\mathrm{without}\;\mathrm{CP}\times 100\%$$

### 2.5. Measurement of Quality Indexes

After treatment with CP, the frozen samples were thawed in a 4 °C refrigerator for 12 h. Then, the quality indexes of the samples were measured. For unfrozen samples, the quality indexes were directly measured after CP treatment. For each quality index at a specific temperature, six samples from the same batch and with the same dimensions were treated with CP to ensure the replicability of the results.

The color of the samples was measured using a portable colorimeter (CR-400, Konica Minolta (China) Investment Ltd., Shanghai, China). The indexes L* (lightness), a* (redness/greenness), and b* (yellowness/blueness) were measured.

After treatment with CP, 5 g of each sample was placed in a homogeneous bag containing 45 mL of distilled water. The samples were homogenized in the homogenizer. Afterward, the pH was measured using universal indicator paper (Hangzhou Shisan Science Co., Ltd., Hangzhou, China).

The samples were weighed, sealed in retort pouches, and immersed in a 75 °C water bath for 90 min. Then, the samples were cooled in an ice–water mixture. The cooking loss was determined via Equation (4):

(4) $$\mathrm{weight}\;\mathrm{loss}\;=\;\left(w_{0}-w_{1}\right)/w_{0}\times \;100\%$$

where *w*_0_ and *w*_1_ are the weights of the samples before and after cooking, respectively.

After measuring the cooking loss, the cooked beef samples were stored in a 4 °C refrigerator for 22 h. Then, the samples were cut perpendicularly to the direction of the muscle fibers using a small-scale shear blade (TA52, Brookfield AMETEK, Inc., Middleborough, MA, USA). The maximum shear force (g) for a 2-cm-deep cut was measured using a texture analyzer (CTX, Brookfield AMETEK, Inc., MA, USA) with a crosshead speed of 1 mm/s.

### 2.6. Statistical Analysis

Single-factor analysis of variance and Duncan’s test were used to compare the significant differences among the means of the results at *p* < 0.05. The statistical analysis software used was SPSS (Version 26, Chicago, IL, USA).

## 3. Results and Discussion

### 3.1. Concentrations of Substances

The ROS and RNS in CP exhibit strong antimicrobial activity [24]. The primary reactions in CP are as follows [33]:

(5) $$\mathrm{NO}+O_{3}\to {\mathrm{NO}}_{2}+O_{2}$$

(6) $${\mathrm{NO}}_{2}+O_{3}\to {\mathrm{NO}}_{3}+O_{2}$$

(7) $${\mathrm{NO}}_{2}+{\mathrm{NO}}_{3}+M⇋N_{2}O_{5}+M$$

In CP, NO reacts with O_3_ to form NO_2_ and is subsequently oxidized to NO_3_. Moreover, high-valence NO_x_ of N_2_O_5_ is formed.

In the current study, the concentrations of typical ROS ozone and typical RNS NO_x_ in the effluent CP gas were indirectly indicated by the concentrations of these substances in PAW. The NO_x_ in the CP dissolved into distilled water, forming long-lasting NO_3_^−^ and NO_2_^−^, as shown in Equations (8) and (9) [34].

(8) $${\mathrm{NO}}_{2}+{\mathrm{NO}}_{2}+H_{2}O\to {\mathrm{NO}}_{2}^{-}+{\mathrm{NO}}_{3}^{-}+2H^{+}$$

(9) $$\mathrm{NO}+{\mathrm{NO}}_{2}+H_{2}O\to 2{\mathrm{NO}}_{2}^{-}+2H^{+}$$

**Figure 3:**
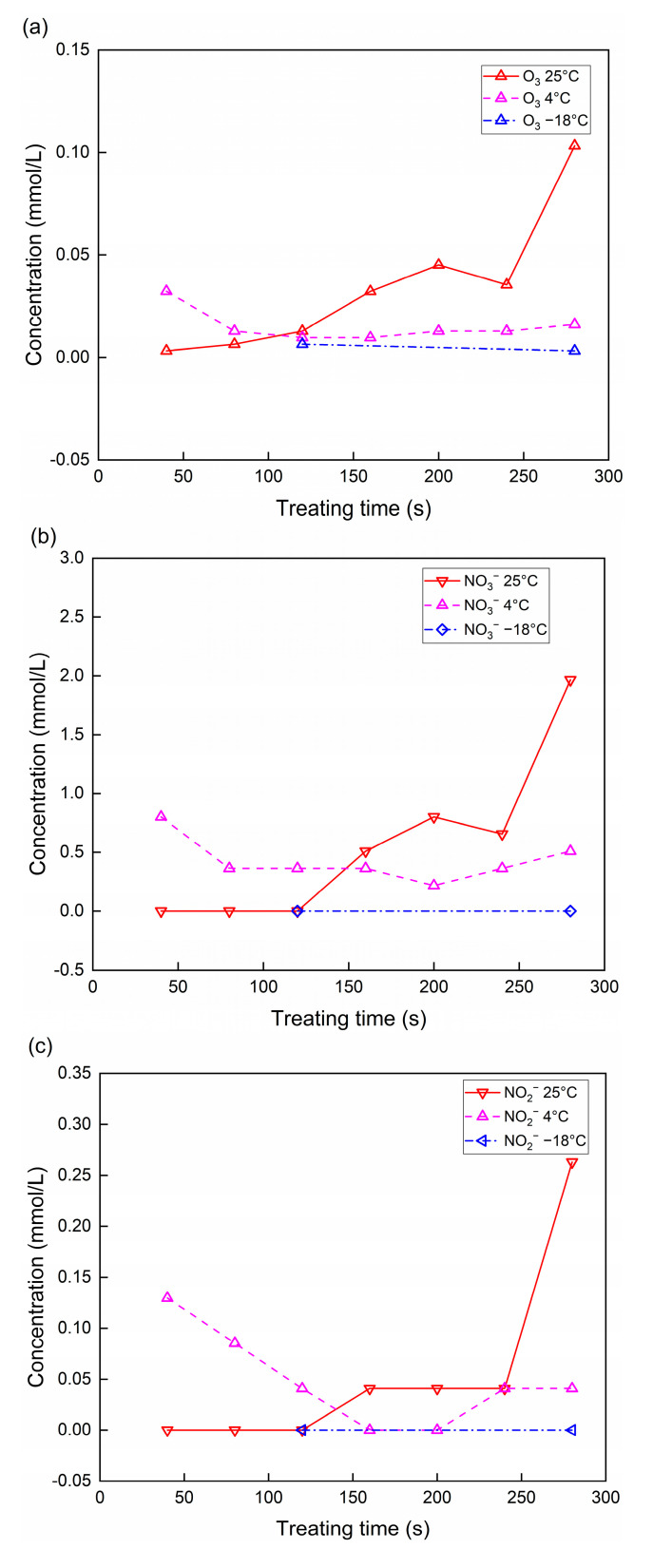
Concentrations of substances in PAW at different temperatures and treatment times. (**a**) O_3_; (**b**) NO_3_^−^; (**c**) NO_2_^−^.

The substances in the PAW at different temperatures are shown in Figure 3. At 25 °C, the substances in the PAW accumulated with the discharge time, resulting in increased concentrations. Then, the substances in the PAW reached a plateau between 160 s and 240 s and started to increase at 280 s. The current results show that the concentrations of O_3_, NO_3_^−^, and NO_2_^−^ in PAW at 25 °C were higher than those at −18 and 4 °C. At −18 °C, the concentrations of the above three components in the PAW were close to zero. At 4 °C, the O_3_ and NO_3_^−^ in the PAW remained at a stable level with time. The concentration of NO_2_^−^ decreased from 40 s to 160 s and started to increase at 240 s.

The trends of the concentration changes of the substances in PAW indicate that the reactive species in CP increased first, reached a plateau, and increased again at 25 °C. At 25 °C, the increase in O_3_ in PAW indicated an increase concentration of O_3_ in the CP, resulting from the continuous air supply. This is consistent with the research conducted by Park et al. [35]. The low concentrations of substances in PAW indicate that the ROS and RNS concentrations in CP under an environment below the freezing point of water might be lower than those under an environment above the freezing point of water. The decrease in NO_3_^−^ and NO_2_^−^ in the PAW with the decreasing temperature was attributed to the decrease in NO_3_ and NO_2_ in the CP. This resulted from the decreasing rate constants of these reactions (Equations (5)–(7)) with the decreasing temperature. As a result, the generation of reactive species in CP might be suppressed below the freezing point of water [33]. At 4 °C, the O_3_ in the CP remained at a stable level over time, while the RNS fluctuated over time. A treatment time of 240 s was used as the bactericidal treatment time in the current research. At this time, the reactive species in the CP exhibited a plateau at 25 °C, a stable level at 4 °C, and a relatively low level at −18 °C.

### 3.2. Bacterial Inactivation

**Figure 4:**
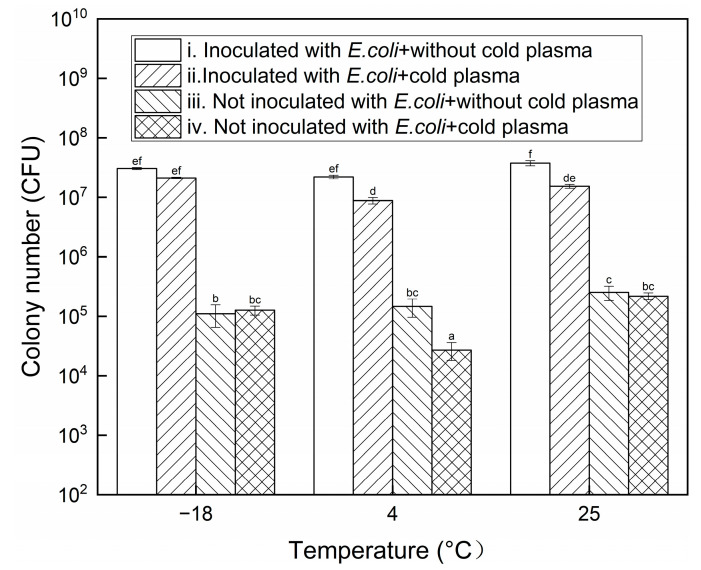
Bacterial colony numbers under different conditions (different letters above bars indicate significant differences among different conditions, *p* < 0.05).

In the current research, the beef placed beside the electrodes was treated with CP. The bacterial inactivation results are shown in Figure 4 and Table S1 in the Supplementary Materials. The bactericidal efficacy of CP at 4 °C was the highest and that at −18 °C was the lowest. The lower inactivation rate at −18 °C might result from the low concentrations of reactive species in CP at this temperature, as described in Section 3.1. Although the concentrations of the substances at 4 °C were lower than those at 25 °C, the bactericidal efficacy was higher than that at 25 °C. At this temperature, the inactivation of the bacteria when the beef was not inoculated with *E. coli* was especially notable. The discrepancy between the inactivation effect and the substance concentration might be attributed to the significant effect of short-lived species in the inactivation process compared to long-lived reactive species [33]. Constrained by the equipment and budget, the short-lived species were not measured in the current study. The colony numbers of the samples not treated with CP and inoculated with *E. coli* or not showed no notable difference between 25 °C and −18 °C, indicating that freezing had no significant effect on the survival of the bacteria.

Previously, researchers have also conducted experiments on the bactericidal effects of CP against pathogens at cold chain temperatures. The experiment conducted by Chen et al. [33] showed that, at −20 °C, 10 min of treatment with CP generated by a coaxial DBD device achieved a 1.1 log reduction in *Salmonella typhimurium* in 1 mm of ice, which was slightly higher than the result of the current study. This might have resulted from the shorter treatment time in the current study and the rough surface of the beef. In addition, the disinfection effect of CP against a dried pseudovirus attached to a plastic flake at −20 °C showed a 1.9 log reduction [36], which was attributed to the simpler structure of the virus compared to that of the *E. coli* used in the current study. Furthermore, the previous research on pathogens was conducted on non-food carriers, differing from the current study, which used beef as the bacteria carrier.

### 3.3. Quality Index

**Figure 5:**
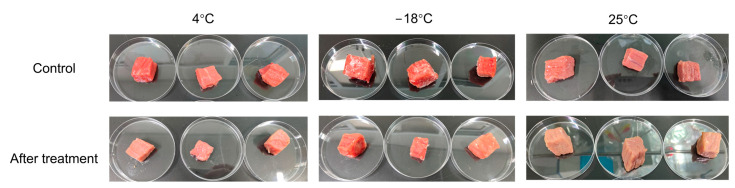
Appearance of the control group beef and groups treated with indirect CP at different temperatures.

The appearance of the beef treated with and without CP is shown in Figure 5. The results of the quality indexes of the color, pH, shear force, and cooking loss for beef treated with and without CP are shown in Table 3. No significant differences existed in the L* of beef treated with and without CP at three typical temperatures. For the a* at 4 °C and 25 °C, the samples treated with CP showed significantly lower values. The reactive species in the CP reacted with the myoglobin in the beef and oxidized the pigment and lipids [24]. At −18 °C, there were no significant differences in the a* between the samples treated with CP and the untreated samples. One possible explanation is that insufficient reactive species were produced at lower temperatures, as indicated in Section 3.1. Another possible reason is that the ice on the surface of the beef inhibited the oxidation effect of the reactive species. For the index b*, samples treated with CP showed significantly lower values at 4 °C. However, at 25 °C and −18 °C, there were no significant differences in the b* of the samples treated with and without CP. Regarding the pH, shear force, and cooking loss, CP showed no significant influence at the three typical temperatures. The shear force and cooking loss at 4 °C were the lowest compared to the other two temperatures. Moreover, at 4 °C, CP showed the highest bactericidal efficacy with a minor influence on the a* of the beef, indicating that this was the most appropriate condition for the storage and sterilization of beef in this research. The investigation conducted by Moutiq et al. also showed that CP had no negative effect on the pH of chicken breast [23]. The hardness of *Trachinotus ovatus* also showed no significant change after treatment with CP without further storage [20].

## 4. Conclusions

In the current study, the bactericidal inactivation effect of CP treatment in beef was evaluated. The concentration of reactive species in CP was the highest at 25 °C and the lowest at −18 °C. This resulted in the higher bactericidal efficacy of CP at 25 °C, reaching 59.5% against *E. coli* on beef. The bactericidal efficacy of CP against *E. coli* on beef at −18 °C was the lowest, at 30.5%.

The CP negatively affected the a* of the beef at 25 and 4 °C. However, this side effect was avoided at −18 °C. CP did not significantly influence the pH, shear force, or cooking loss of the beef. At 4 °C, CP showed the highest bactericidal efficacy with a minor influence on the beef quality, indicating that 4 °C was the most appropriate temperature for the storage and sterilization of beef under the three conditions considered in this research. Furthermore, indirect CP shows promising bactericidal effects for industrial food applications, with the potential to maintain food safety if its bactericidal efficacy at −18 °C can be enhanced in future research. Overall, indirect CP was able to disinfect food contaminated with *E. coli* at 4 and −18 °C, with a relatively low impact on the food’s quality.

The current research will be helpful in reducing pathogen contamination in the food industry, especially in the cold chain. The application of CP together with refrigeration technology in the cold chain will help to maintain food quality and reduce food waste.

## Figures and Tables

**Table 1:** Color of beef treated with and without direct CP treatment.

	L*	a*	b*
Control group	41.01 ± 1.31 ^a^	13.72 ± 1.13 ^b^	7.07 ± 1.13 ^a^
Direct CP treatment	42.68 ± 1.00 ^ab^	8.81 ± 0.93 ^a^	8.41 ± 0.97 ^ab^

Different superscripts show significant differences among different conditions within each column (*p* < 0.05).

**Table 2:** The correlation between the absorbance at specific wavelengths and the concentrations of active substances in PAW.

Active Substance	Characteristic Wavelength/nm	Correlation between Absorbance A (Abs) and Concentration C (mmol/L)
O_3_	260.0	A = 0.31·C [31]
NO_3_^−^	302.1	A = 0.00686·C + 0.00451

**Table 3:** Quality index values of beef treated with and without CP at different temperatures.

	L*	a*	b*	pH	Shear Force (g)	Cooking Loss (%)
25 °C control	43.67 ± 0.36 ^ab^	16.04 ± 0.73 ^bc^	9.17 ± 0.42 ^ab^	6 ± 0 ^a^	4115 ± 179 ^b^	41.7 ± 0.3 ^a^
25 °C CP	44.86 ± 0.93 ^b^	9.90 ± 0.57 ^a^	9.38 ± 0.58 ^b^	6 ± 0 ^a^	4128 ± 189 ^b^	42.0 ± 0.2 ^a^
4 °C control	50.46 ± 1.61 ^c^	21.43 ± 0.89 ^e^	15.37 ± 0.45 ^d^	6 ± 0 ^a^	3151 ± 219 ^a^	39.2 ± 2.0 ^a^
4 °C CP	48.36 ± 0.93 ^c^	17.97 ± 0.82 ^cd^	12.65 ± 0.45 ^c^	6 ± 0 ^a^	3100 ± 355 ^a^	35.8 ± 4.7 ^a^
−18 °C control	44.08 ± 1.00 ^ab^	19.66 ± 0.70 ^de^	12.61 ± 0.44 ^c^	6 ± 0 ^a^	3994 ± 127 ^b^	49.4 ± 0.3 ^b^
−18 °C CP	43.18 ± 1.01 ^ab^	18.33 ± 1.15 ^cd^	11.48 ± 0.83 ^c^	6 ± 0 ^a^	4486 ± 225 ^b^	48.5 ± 0.6 ^b^

Different superscripts show significant differences among different conditions within each column (*p* < 0.05).

## Data Availability

The original contributions presented in the study are included in the article/Supplementary Materials, further inquiries can be directed to the corresponding author.

## Supplementary Materials

The following supporting information can be downloaded at: https://www.mdpi.com/article/10.3390/foods13172846/s1, Table S1. Bactericidal efficacy of CP at different temperatures. Figure S1. Applied Voltage and discharge current of CP.
