# Supplementary material for: Experimental Investigation of Bacterial Inactivation of Beef Using Indirect Cold Plasma in Cold Chain and at Room Temperature
Source: Foods. 2024 Sep 7;13(17):2846. doi: 10.3390/foods13172846 (PMC11395448; doi:10.3390/foods13172846)
Supplement: Supplementary file 1 [file foods-13-02846-s001.zip › foods-3096424-supplementary.pdf]

Table S1. Bactericidal efficacy of CP at different temperatures

	**-18 °C**	**4 °C**	**25 °C**
Inoculated with *E.coli*	30.5 ± 2.2 %[b]	60.1 ± 4.9 %[c]	59.5 ± 3.1 %[c]
Not inoculated with *E.coli*	0[a]	81.4 ± 6.3 %[d]	56.9 ± 11.6 %[c]

Different superscripts show the significant difference among different conditions ($p<0.05$).

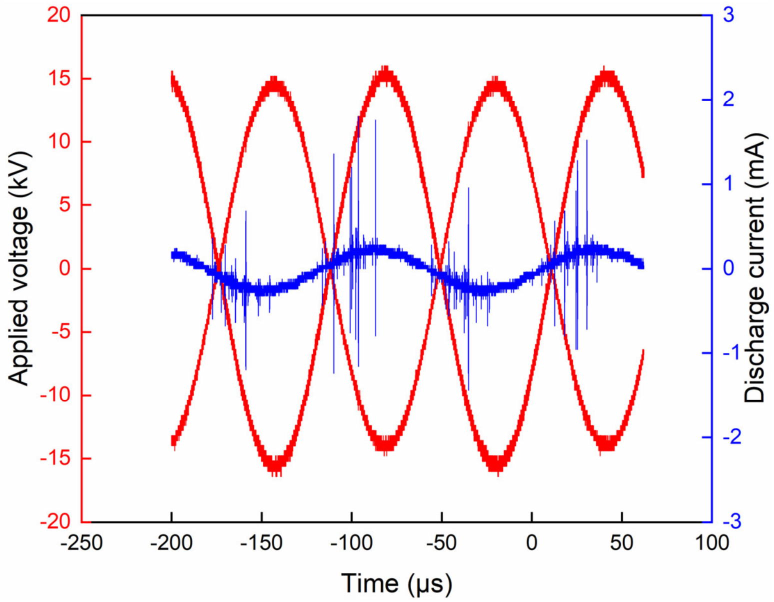

Figure S1. Applied Voltage and discharge current of CP.
